# Caesarean section epidemic in India: Is private sector to blame? A multivariate logistic regression analysis of the National Family Health Survey

**DOI:** 10.1371/journal.pone.0352156

**Published:** 2026-07-06

**Authors:** Manoj Kumar, Akshat Kumar Singh

**Affiliations:** Department of Economics, Banaras Hindu University, Varanasi, India; All India Institute of Medical Sciences - Raipur, INDIA

## Abstract

**Background:**

C-sections (CS) can be lifesaving in certain medical situations, but their prevalence has surged beyond recommended levels globally, raising concerns about inappropriate medical interventions and healthcare delivery quality.

**Objectives:**

The study explores whether medical conditions alone determine C-sections or if socio-economic and institutional factors (i.e., Private/Public) also play a significant role.

**Method:**

Using WHO and World Bank data, the relationship between C-section rates and income was analysed at the global level. Furthermore, at the national and state levels in India, the same analysis was conducted using data from the Ministry of Statistics and Programme Implementation and NFHS-5. Additionally, utilising the Birth recode datasets of NFHS-4 and 5, multivariate logistic regression was performed with C-section as the outcome variable and socio-economic and institutional variables, such as place of residence, levels of education, wealth index, and place of delivery, as predictor variables.

**Results:**

Throughout the analysis, we found the institutional setting as the most significant influencing factor of CS rates, at both the national (OR: 4.11, 95% CI 3.98–4.24; NFHS-5) and state level for Bihar (OR: 16.19, 95% CI 13.76–19.05; NFHS-5), Uttar Pradesh (OR: 8.62, 95% CI 7.84–9.47; NFHS-5), Tamil Nadu (OR: 3.08, 95% CI 2.70–3.52; NFHS-5) and Andhra Pradesh (OR: 4.16, 95% CI 3.40–5.09, NFHS-5). On the contrary, we found that socioeconomic factors influenced the likelihood of CS only in states where medical infrastructure was lacking, indicating that socioeconomic factors are not directly responsible for CS rates; they are complicit only in determining institutional access. Additionally, an inverted U-shaped relationship was found between national per capita income and CS rates, indicating global inequality in the quality of healthcare. Within India, this relationship increasingly mirrors the global trend, possibly due to disparities in healthcare access and quality.

**Conclusion:**

Our analysis confirms that the increase in CS rates is not solely caused by medical conditions, but is also significantly influenced by non-medical factors, particularly institutional factors. Economic incentives strongly drive private healthcare providers to prefer CS deliveries. The results suggest the need for targeted policy interventions to mitigate perverse incentives for private facilities and enhance public medical infrastructure, particularly in underserved regions.

## 1. Introduction

*“Macduff was from his mother’s womb untimely ripped.” (Act 5, Scene 8)*

*~ Macbeth, William Shakespeare* [1]

### 1.1. What is CS, & Why is it performed?

The American College of Obstetricians & Gynaecologists defines Caesarean Section (CS) or Caesarean birth as a surgical delivery of the baby through a surgical cut or incision in a woman’s abdomen and uterus [2]. Childbirth is inherently a natural process; however, various complications may arise during birth that would make a normal birth risky for both the mother and the child. In such a situation, CS acts as a prudent solution [3]. More recently, however, CS are also performed for the convenience of the mother, called elective CS, which are fairly common in South America and the United Kingdom [4,5].

### 1.2. The evolution of CS around the world

With the development of technology and a better understanding of human anatomy, CS became increasingly common in advanced economies starting in the 1930s [6]. Since then, CS has been a common procedure in most parts of the world, and is now an extremely safe procedure, in some cases even safer than vaginal birth [7]. Birth, which for aeons occurred at homes with assistance from family elders and midwives, became a hospital matter, which some have defined as the *Medicalisation* of birth [8]. The benefits of increased CS are visible retrospectively, as CS were invaluable in case of severe complications, where both the mother’s and child’s lives were at risk. The rise in the availability of CS has been linked with the global fall in the Neonatal Mortality Rate (NMR) & Maternal Mortality Rate (MMR) [9].

As significant investments in medical infrastructure were made in developing countries, CS rates began to increase, and today, most of the middle-income countries have surpassed the developed world in CS rates. However, the least developed countries continue to have very low rates of CS, as medical facilities, skilled medical professionals and the capacity to use these facilities remain largely absent, which is responsible for the higher-than-average NMR and MMR rates [10].

**Fig 1:**
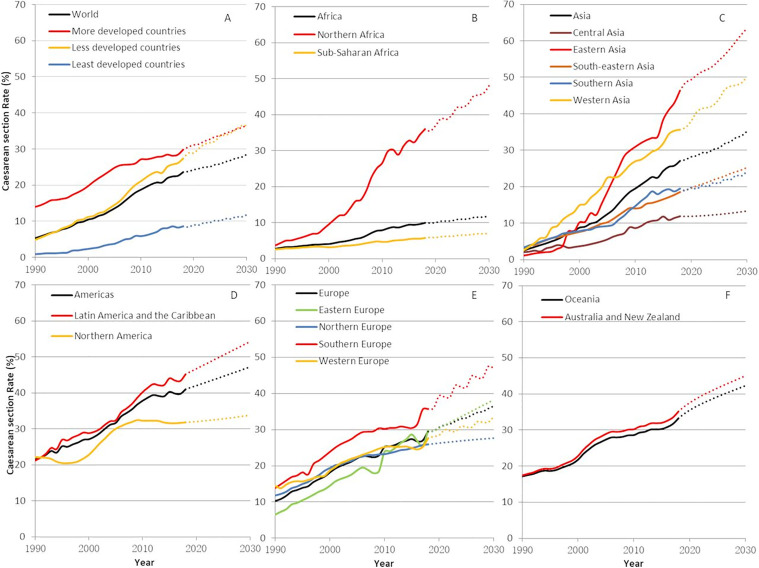
Global trends in C-section rates, 1990-2020*. Source: * Reprinted under Open Access CC BY-NC license [11].

**Fig 2:**
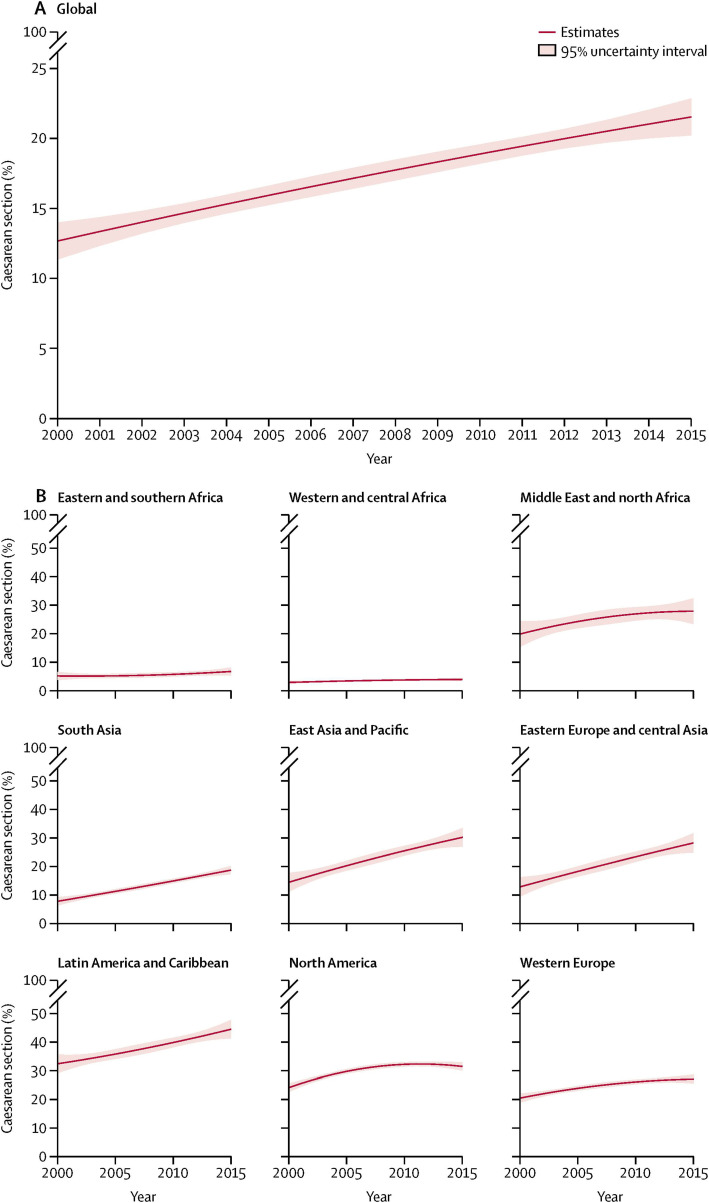
Regional trends in C-section rates, 1990-2018^. Source: ^ Reprinted with permission from Elsevier [12].

Over the past 30 years, for which we have more detailed and granular data, CS rates have increased globally, regardless of level of development, income, region, or any other factor. Fig 1 illustrates this uniform global rise. Today, the highest CS rates are in South America and the Caribbean, while the lowest are in Sub-Saharan Africa [11]. CS rates in much of the developed world have increased at a much lower rate, and more recent data indicate stagnation in CS rates over the past 10–15 years, due to increased awareness and stronger medical guidelines on overuse. Fig 2 shows the apparent arrest in the growth rate of CS in Western Europe, Scandinavia and North America [12].

### 1.3. The concern with increasing CS rates

The astronomical increase in CS rate is of major concern, as the age-old adage warns, “*the excess of anything is bad”.* This trend in India reflects a pattern observed internationally, where CS rates tend to follow an inverted U-shaped relationship with per capita income. As countries develop economically, CS rates typically increase alongside rising incomes, eventually stabilizing or declining as health systems mature and implement more evidence-based guidelines [13,14] India currently appears to be positioned on the ascending portion of this trajectory, with CS rates rising substantially as economic development progresses, particularly within urban areas and private healthcare facilities. WHO, after extensive deliberation and an ecological study of birth at the population level in 1985, concluded that any increase in CS rate above 10–15% is not associated with a reduction in NMR & MMR [15]. A follow-up study in 2016 found the same results [16]. Although this 15% CS rate has been widely cited as a benchmark, WHO has clarified that it does not represent the ideal rate, as other factors, particularly morbidity, were not considered in the study [17]. Another study found that CS rates up to 19% were inversely correlated with NMR & MMR [18]. Thus, it could be asserted that the ideal rate of CS is between 10–19%. However, with CS rates in most countries now exceeding the ideal rate, it has become a global public health concern.

Even though CS is a routine surgery, they are still considered a major surgery and carries significant risks such as infection, uterine rupture, haemorrhage and other complications [19]. CS have also been linked with placenta previa and increased odds of miscarriage and stillbirth in future pregnancies [20]. Additionally, CS also increases the likelihood of needing CS in future pregnancies, compounding the associated risks [21]. Studies also show an elevated risk of Postpartum Depression following CS deliveries [22]. Recent evidence indicates CS can have a long-term impact on the health outcomes of children, including increased risk of Asthma [23], alleviated risks of allergy, autoimmune diseases and hindered postnatal immune development [24], and higher chances of obesity and type II diabetes [25]. The rise in unnecessary CS around the world has led some medical journalists to call it the “*C-section Epidemic”* [26].

From a monetary perspective, studies worldwide have found that CS tend to be more expensive than vaginal deliveries, particularly in private hospitals [27,28]. A similar study in India estimated that the cost of CS, especially in private hospitals and nursing clinics, is much higher. They also found that the cost burden was highest for the low-income families, who spent 10% of their annual income for a CS at a government facility and 27% at a private facility [29]. Furthermore, another study using NFHS data revealed that CS deliveries were not only more expensive but also increased the extent of distress financing, with over 15% of CS requiring distress financing; the rate was higher in Private facilities at 27% [30]. A WHO study conducted in 2010 estimated the global number of unnecessary CS is around 6 Million, with a corresponding cost of US$ 2.32 Billion annually [31]. Bhatia et al. (2020), based on their analysis of NFHS-4, found the number of avoidable CS to be 1.83 Million, and they estimated a total saving of US$ 320.60 Million if private institutions in India adhered to the 15% optimal rate of CS [32].

There is also a resource allocation concern as CS rates vary significantly within the country and between socioeconomic groups, with some regions and groups having much lower rates of CS than recommended and, by extension, having higher NMR & MMR [33]. In India, the variation is stark, with states like Telangana having CS rates closer to 60% (4 times the recommended) and states like Bihar where rates are less than 10%. Thus, in a country where resources are thinly stretched, unnecessary CS add an extra burden on the healthcare system and direct resources away from those who actually need them.

### 1.4. The causes of increasing CS rates

In addition to the medicalisation of birth, other significant factors also contribute to the increasing rate of CS. The mean age of childbearing has increased globally, and in India, the share of women having their first birth below the age of 30 has decreased from 43% in 1993–94–30% in 2019–21 [34,35]. Medical literature indicates that the likelihood of needing a CS increases with the increase in maternal age at birth, particularly after age 30 [36]. Another contributing factor is the increase in obesity and Body Mass Index (BMI) among women worldwide, including India [37]. A study found that 40% of women in India were obese, with 50–60% of women between the ages of 30–49 being obese [38]. Obesity and high BMI are linked with a higher likelihood of needing a CS [39].

The economic driver of increasing CS rates is theoretically explained by the concept of Physician-Induced Demand, propounded by Robert G. Evans [40]. He demonstrated that information asymmetry between physician and patient allows the physician to exert “*non-price influence on the demand of his own services*” [40]. This implies that physicians have perverse incentives to induce demand, influencing the quantity or the price of services being offered [41]. Specially, it has been shown that Physicians can exploit information asymmetry when faced with an income shock by providing excessive or more expensive care to maintain their earnings. For instance, Gruber and Owings (1994) found that the fall in the fertility rate in the US from 1970−1982 led to an increase in income pressure for Obstetrics and Gynaecologists (OB-GYN), which led them to substitute normal deliveries with CS, which are more expensive, thus inducing demand for CS. They found that a 13.5% fall in fertility between 1970−82, led to a 240% increase in CS deliveries [42]. This evidence is crucial in establishing monetary incentives as a major driver of increasing the CS rate.

Looking at the incentives at play, a qualitative study found that various factors incentivise doctors and hospitals to prefer CS over vaginal delivery. The most significant among these were monetary incentives, which acted to increase CS rates in two ways. First, patients pay more for CS, due to additional charges for operating theatres, anaesthetists and prolonged hospital stays. Respondents asserted that hospitals generate 30–50% more revenue through CS compared to vaginal delivery. Second, CS takes a much shorter time to perform compared to vaginal deliveries. This allows hospitals/OB-GYNs to maximise the volume of patients that they can accommodate, further increasing profitability. The longer time concern is compounded by the fact that the number of OB-GYNs in India is far below the global average. Additionally, the existence of a large number of solo practice nursing clinics, where for a vaginal delivery the doctor would need to be available 24/7, creates a strong incentive to perform CS. The study also found an immense shortage of skilled birth assistants (SBAs), particularly trained midwives, who could have alleviated the load on the OB-GYNs [43].

Viewed through the lens of the Social Determinants of Health, as set out by the WHO Commission on Social Determinants of Health, the conditions in which women “are born, grow, live, work and age” shape both their exposure to obstetric risks and their negotiating power within the health-care system. Socio-economic gradients in India illustrate this: NFHS-5 data show that caesarean deliveries are far more common among wealthier women giving birth in private facilities than among poorer quintiles using public services, even after controlling for clinical indications.

### 1.5. Socio-cultural factors

Several social and cultural factors contribute to the rising CS rates in India. Many communities now view CS as modern and safer than vaginal delivery, often linking it to a higher social status. Fear of labour pain motivates preferences for surgical delivery, especially among urban, educated women who seek “painless birth” options in private hospitals. Family involvement significantly influences delivery decisions. Husbands and in-laws often prioritize convenience and believe surgical birth ensures better outcomes for mother and child. Cultural practices around auspicious timing for births, based on astrology, frequently result in scheduled CS [43]. Concerns about care quality and pain management in public hospitals drive families toward private providers who promote elective procedures [44]. These social preferences, combined with healthcare system incentives, sustain the upward trend in CS use across different population groups.

## 2. Research gap

Literature on the prevalence of CS in India has predominantly approached the issue from a medical perspective, focusing on factors such as maternal age, BMI, and nutrition. While some studies have considered the socioeconomic and institutional determinants of CS, most have been limited to the NFHS-4. Only a few studies have extensively examined both socioeconomic and institutional factors using NFHS-5. Roy N., et al. (2021) identified a myriad of factors influencing the rising CS rate; their analysis of NFHS-4 & NFHS-5 found that the CS rates were higher in southern states compared to the northern states, and also corroborated other research that both increasing age and BMI are major factors influencing the rise in CS. They reported CS rates at the national level and for only a few selected states, but their analysis of factors determining the CS rate was confined to the NFHS-4 [45]. Pandey A.K., et al. (2023) found that the CS rates in public institutions decreased from 15.9% in 1999 to 14.3% in 2020, while in Private institutions, it increased from 24.7% to 47.4% over the same period. However, they only analysed the role of socioeconomic and institutional factors influencing CS rates at the national level and did not explore the underlying disparities between the states [46]. Mohan et al. (2023) identified changing medical factors, such as increasing maternal age at birth and BMI, along with socioeconomic factors, as key determinants of the increasing CS rate. They noted that CS rates increased significantly between NFHS-4 & NFHS-5. They also found extreme disparities in CS rates between states, as well as more pronounced disparities between the public and private sectors. On this basis, they concluded that the place of delivery, whether public or private, is the most significant factor influencing CS [47].

Given this background, the present study aims to provide a comprehensive analysis of CS rates and influencing factors at both the national and state levels. In the study, four states, namely Tamil Nadu, Andhra Pradesh, Bihar and Uttar Pradesh, have been considered. It explores how factors such as income level, access to medical facilities and infrastructure influence CS rates. Additionally, this study also analyses the determining factors responsible for the increasing CS rates in the country. A key objective is to address the conundrum of whether it is Private hospitals that are responsible for high rates of CS, or whether it is socio-economic factors that induce demand for CS, and the private sector just fulfils this demand.

This study adopts a theoretical lens that highlights the role of incentives and informational asymmetry, which enable private institutions to influence CS rates. It examines cost differences between CS and vaginal deliveries across public and private hospitals. It draws on qualitative literature to demonstrate how personal and institutional motivations create conditions that enable the private sector to exert undue influence over delivery decisions. Based on this framework, the study addresses two research questions: first, what are the key factors influencing CS rates in India, and how do these factors vary across different states and socioeconomic groups? Second, how do private sector practices and cost differences between public and private institutions shape the prevalence and utilisation patterns of CS in India?

## 3. Data and methodology

National Family Health Surveys (NFHS) 4 and 5’s Birth Recode datasets were used in the study. NFHS is the Indian version of the DHS (Demographic Health Survey). It is a nationally representative survey conducted by the Ministry of Health and Family Welfare (MHFW) and the International Institute for Population Studies (IIPS) [48]. NFHS captures key information regarding women’s and child’s health, reproduction, fertility and other sex and health-related factors. The Birth Recode dataset of the NFHS includes one record for each child born to interviewed women aged 15–49 years in the five years preceding the survey. Thus, the dataset provides information on children aged 0–59 months born to women aged 15–49 years.

Descriptive analysis was performed on certain key variables to determine the CS rate and out-of-pocket expenditure. Furthermore, country-wise per-capita income and CS rate data from the World Bank and the World Health Organisation were used to analyse the relationship between the CS rate and income level. Similarly, for the state level, we used per capita income reported by the Ministry of Statistics and Programme Implementation and the CS rate calculated from NFHS-5 to analyse the relationship between the CS rate and income level.

Additionally, multivariate logistic regression were performed to determine the relationship between socio-economic and institutional factors and CS rates at the national and state levels. The outcome variable for the study was the CS rate, which in the NFHS is stored as a dummy variable as a response to the question “*Was the baby delivered by caesarean section, that is, did they cut your belly open to take the baby out?”*. Where birth through CS is coded as 1 and coded as 0 for vaginal delivery. In the study, Place of residence, Level of education, Wealth index, and Place of delivery were chosen as predictor variables, representing socioeconomic and institutional factors.

The regression analysis across Bihar, Uttar Pradesh, and Andhra Pradesh demonstrated a rigorous and well-specified modelling strategy that underpins the reliability of the results. For example, the models achieved high discriminatory power, as shown by the Area Under the ROC Curve (AUC) values: 0.8362 in Bihar, 0.8147 in Uttar Pradesh, and 0.7144 in Andhra Pradesh, indicating a strong ability to correctly classify caesarean versus non-caesarean deliveries. The Pseudo R² statistics ranged from 0.1196 to 0.2672, reflecting acceptable explanatory power in logistic regression. Careful attention was paid to model specification and functional form. The negative sign on the squared predicted values suggests that the relationship between predictors and the outcome is curvilinear rather than strictly linear, which is consistent with including quadratic terms to capture any nonlinear effects. The most important variables and those contributing significantly to the model were retained after systematic testing. When additional interaction variables were included, the Wald chi-square statistic declined, indicating that these interactions did not improve model fit and were therefore excluded. Robust estimation methods were applied throughout. Robust standard errors with the cluster option were used to account for potential heteroscedasticity and clustering in the survey data. Additionally, formal tests indicated no significant heteroscedasticity in the models.

To further address concerns regarding potential endogeneity of the independent variables, we conducted the Rivers-Vuong (1988) test. This procedure is an extension of the Durbin-Wu-Hausman approach adapted for non-linear models such as logistic regression. In this test, endogeneity is indicated if the instrumented residuals from the first-stage regressions remain in the model and are statistically significant predictors of the outcome variable. In our analysis, all residuals were omitted due to perfect collinearity, implying that the independent variables fully accounted for the relevant variation and did not exhibit additional unexplained associations with caesarean delivery outcomes. This pattern strongly suggests that the regressors are exogenous and that endogeneity is unlikely to bias the coefficient estimates. Accordingly, the results can be interpreted as robust associations between socioeconomic and institutional factors and CS rates.

The functional form of the logistic regression is given as follows.


$$\begin{array}{c} og\left(\frac{\left(P_{\text{1}}\right)}{\left(\text{1}-P_{\text{1}}\right)}\right)=\beta_{\text{0}}+\beta_{\text{1}}X_{\text{1}}+\beta_{\text{2}}X_{\text{2}}+\beta_{\text{3}}X_{\text{3}}+\beta_{\text{4}}X_{\text{4}}+\;U_{i} \end{array}$$

Here,

P_1_ is the probability of a birth through CS & (1 – P_1_) is the probability of a birth through vaginal delivery

X_1_ is the place of delivery, coded 0 for public institution and 1 for private

X_2_ is the wealth index, coded 1 through 5, denoting five wealth quintiles ranging from poorest to richest

X_3_ is the highest level of maternal education, coded 0 through 3, ranging from no education to higher

X_4_ is the place of residence, coded 0 for rural and 1 for urban

U_i_ is the error term

All statistical analyses were performed in STATA version 18.0. Standard errors for all regressions were specified to be robust, and DHS-specified clusters were used as the unit of analysis. The dataset was used as provided without additional manipulation.

## 4. Results

Birth is a purely medical phenomenon, and so is the determination of whether one should give birth through CS or vaginal delivery. It is often assumed that the decision to perform a CS is based primarily on medical factors; however, our study shows that non-medical factors play a significant role. We found that factors that have less to do with the medical conditions of the child or the mother are the most powerful determinants of whether the birth is through CS.

To begin with, we examined CS rates globally. There could indeed for which we anticipated some variation in CS rates across countries due to differences in women’s health conditions. However, despite these variations, the human body and the process of birth are fairly universal from a medical perspective. As extensive ecological studies from around the world have shown, CS rates above the 10–15% level are not linked with any improvement in NMR or MMR. Thus, the large variations in CS rates across countries cannot be explained solely by medical factors, indicating the influence of other factors in determining CS rates.

### 4.1. Global variation in CS rates and the income determinant

**Fig 3:**
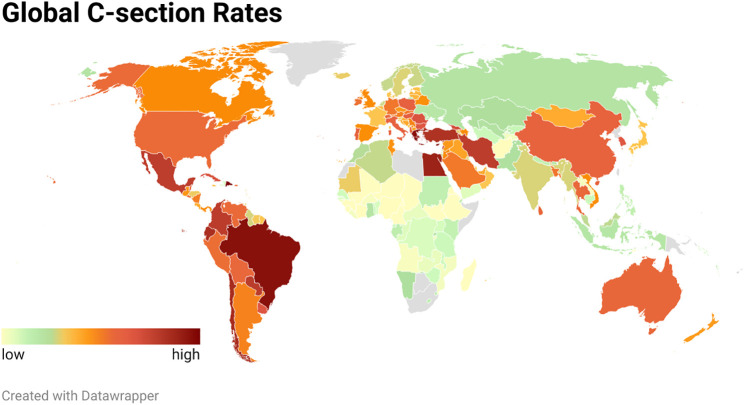
Spatial distribution of C-section rates by country, circa 2020. Source: Created with Datawrapper using World Health Organisation data (CS Rates) [49].

Fig 3 clearly illustrates the substantial variations in CS rates across countries, with notable regional clustering. The CS epidemic is most rampant across South America, followed by Turkey and Egypt. India’s CS rate is relatively low when compared to other countries. There is, however, a huge regional disparity, discussed later in Section 4.2. Additionally, India is experiencing some of the fastest increases in CS rates anywhere in the world.

**Fig 4:**
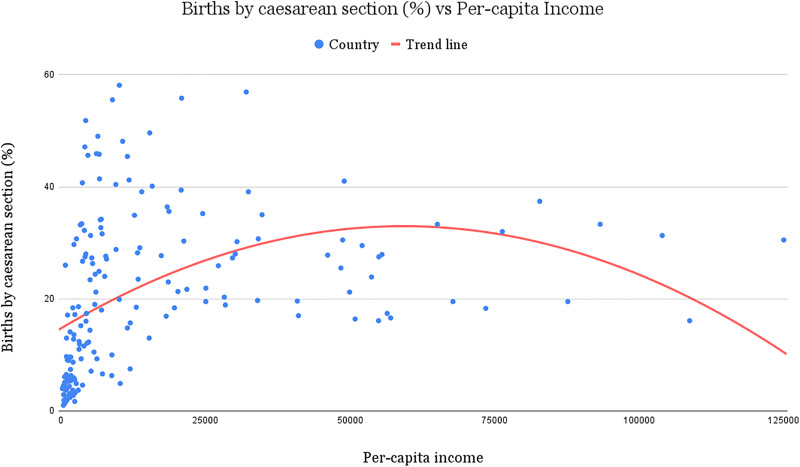
C-section rates by per capita income across countries. Source: World Health Organisation (CS Rates) [49] World Bank (per capita income) [50].

In Fig 4, we examine how income, arguably the most influential socioeconomic factor, affects CS rates. Income can influence CS rates in two ways: first, by determining access to medical facilities, and second, as a proxy for overall socioeconomic affluence, including factors such as education and literacy, which have been linked to higher demand for elective CS due to perceived lower risk and pain [51,52].

As shown, we observe that CS rates vary significantly between countries. Moreover, the trend clearly follows an inverted U-shape. CS rates are lower for countries with very low per-capita income, primarily because of a lack of medical facilities and limited access to skilled birth assistants. As income increases, however, CS rates also increase, but only to a point. Middle-income countries happen to have the highest rates of CS around the world. Brazil, Egypt, China, and Turkey are all middle-income countries with some of the highest CS rates. This is primarily due to almost universal access to medical facilities, but a lack of both stringent regulation and awareness about the concerns of unnecessary CS. High-income countries still have CS rates considerably lower than those of middle-income countries, which is largely due to the higher quality of medical facilities, a strong focus on wellbeing, robust regulation, and increased awareness among women.

### 4.2. National variation in CS rates and the income determinant

**Fig 5:**
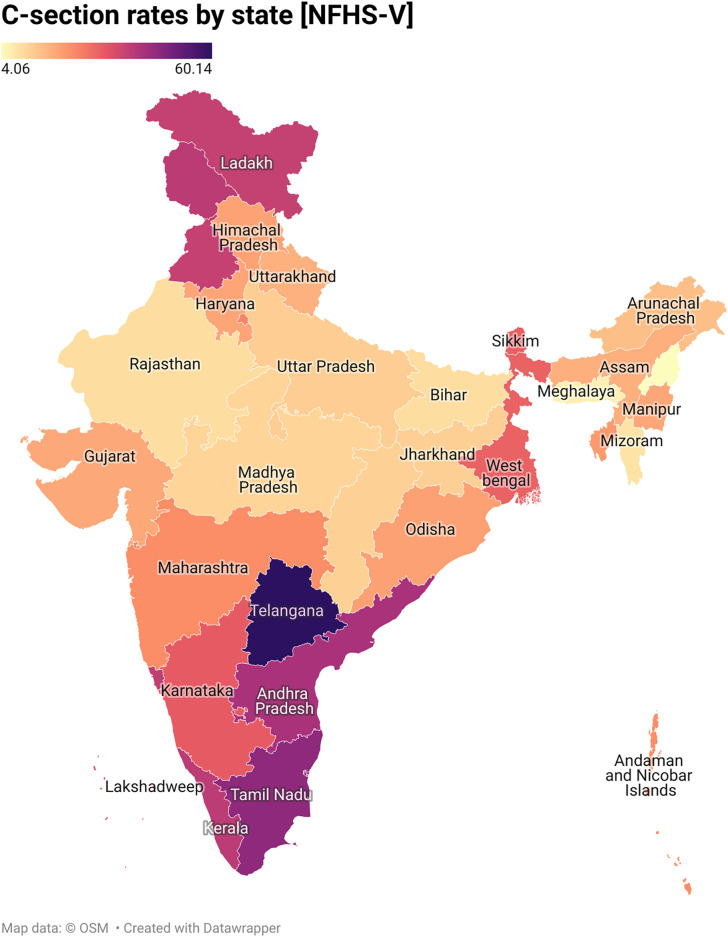
State-level variation in C-section rates in India, 2019–2021. Source: Created with Datawrapper using NFHS-5 data.

We found significant variation in CS rates within India. It is evident from Fig 5 that a divide exists within India, with northern states, particularly those with some of the highest populations, having some of the lowest CS rates. On the contrary, southern states have much higher CS rates, not only when compared to northern states but also at the global level.

**Fig 6:**
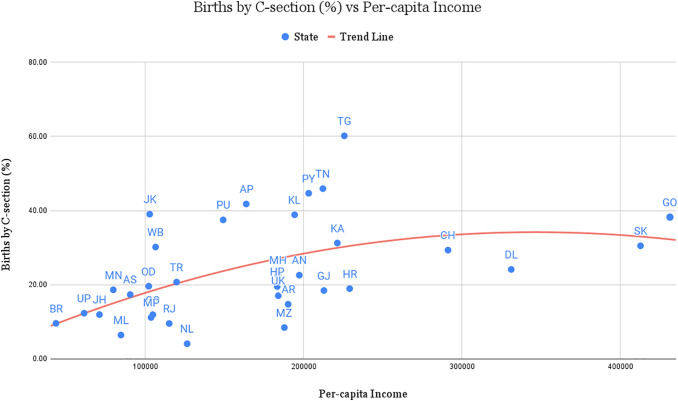
C-section rates by per capita income across states in India. Source: NFHS-5 (CS Rates). Ministry of Statistics & Programme Implementation (Income) [53]. # For state names refer to Appendix 1.

Fig 6 plots the CS rates of states against their per capita income, revealing a similar trend to the global measures. CS rates are lowest for states with lower per-capita income, and as the income level increases, so does the CS rate. This could mostly be explained by higher income being closely linked with better medical facilities and increased access to those facilities. Consistent with the global trend, CS rates increase only to a certain level of per capita income and beyond that, they stagnate and even start to decline. The middle-income states tend to have the highest rates of CS in the country. However, deriving much inference about the CS rate and its linkage with income is difficult due to significant variation in medical infrastructure, socioeconomic conditions, and other influencing factors across these states, albeit these factors themselves are influenced greatly by income levels.

**Fig 7:**
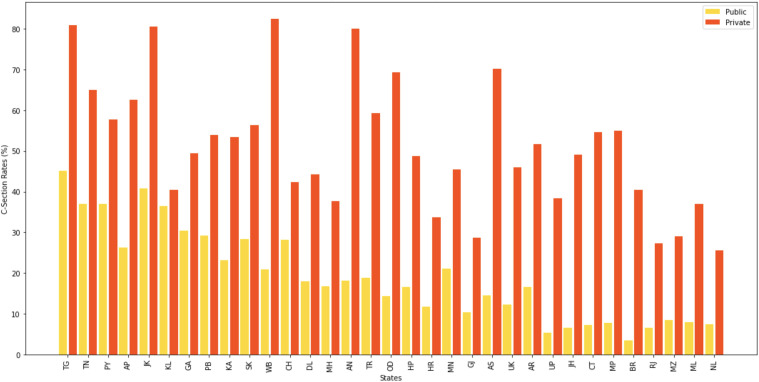
C-section rates by institution type (public vs private) across states. Source: Calculated from NFHS-5.

The CS rates for both the Public and Private facilities are reported for each state in Table 1. There is significant variation in CS rates between states, with the CS rate being lowest in Nagaland and highest in Telangana. States with large populations, such as Bihar and Uttar Pradesh have CS rates closer to 10%. What is interesting, however, is the drastic variation in CS rates in Public and Private facilities the rates in private facilities being greater (*almost double*) than public for nearly all states, as shown in Fig 7. However, there also seems to be a considerable degree of variation in CS rates at public institutions in different states. Looking at it from another perspective, the CS rate in public institutions in states with high CS tends to be equal to or even higher than the CS rates in Private facilities in states with lower overall CS rates. One potential cause for the lower CS rates in public facilities in states like Bihar and UP could be the high patient load, which could lead to rationing, where medical professionals have to allocate their time and effort to a larger number of patients thus even when births within the public facilities require a CS, the procedure might not be performed because of limited medical capacity. The same resources and capacity are not as stretched in states with higher public CS rates, which would mean that even in public facilities, those who require CS are provided with such interventions.

**Table 1:** CS rates by State and Institution type.

State	CS Rate (%)			State	CS Rate(%)		
	Total	Public	Private		Total	Public	Private
**Telangana**	*60.14*	*45.02*	*80.84*	**Himachal Pradesh**	*19.51*	*16.64*	*48.66*
**Tamil Nadu**	*45.89*	*37.00*	*65.05*	**Haryana**	*18.93*	*11.82*	*33.64*
**Puducherry**	*44.65*	*36.92*	*57.64*	**Manipur**	*18.60*	*21.06*	*45.41*
**Andhra Pradesh**	*41.76*	*26.25*	*62.49*	**Gujarat**	*18.42*	*10.30*	*28.63*
**Jammu & Kashmir**	*39.01*	*40.81*	*80.57*	**Assam**	*17.32*	*14.57*	*70.24*
**Kerala**	*38.84*	*36.42*	*40.47*	**Uttarakhand**	*17.02*	*12.28*	*45.96*
**Goa**	*38.21*	*30.37*	*49.35*	**Arunachal Pradesh**	*14.72*	*16.59*	*51.74*
**Punjab**	*37.46*	*29.21*	*53.97*	**Uttar Pradesh**	*12.31*	*5.40*	*38.41*
**Karnataka**	*31.23*	*23.18*	*53.31*	**Jharkhand**	*11.92*	*6.53*	*49.07*
**Sikkim**	*30.48*	*28.38*	*56.34*	**Chhattisgarh**	*11.91*	*7.23*	*54.57*
**West Bengal**	*30.15*	*20.85*	*82.38*	**Madhya Pradesh**	*11.15*	*7.77*	*55.00*
**Chandigarh**	*29.31*	*28.17*	*42.31*	**Bihar**	*9.57*	*3.53*	*40.38*
**Delhi**	*24.11*	*18.06*	*44.24*	**Rajasthan**	*9.53*	*6.55*	*27.39*
**Maharashtra**	*22.68*	*16.81*	*37.72*	**Mizoram**	*8.44*	*8.48*	*29.07*
**Andaman & Nicobar**	*22.56*	*18.14*	*80.00*	**Meghalaya**	*6.41*	*7.99*	*36.99*
**Tripura**	*20.68*	*18.91*	*59.35*	**Nagaland**	*4.06*	*7.39*	*25.54*
**Odisha**	*19.58*	*14.40*	*69.32*	**India**	*19.21*	*13.95*	*47.18*

Source: Calculated from NFHS-5.

### 4.3. Cost differentials in delivery type and institution type

The average Out-of-Pocket Expenditure (OOPE) for each delivery type and institution is presented in Table 2. The OOPE for a delivery also varies significantly depending on the mode and the place of delivery. We find that even in public facilities where healthcare is highly subsidised, the OOPE for caesarean delivery was nearly four times the cost of normal delivery. In the private sector, the OOPE for caesarean was about 2.25 times that of normal vaginal delivery. A starker difference emerges when comparing public and private facilities, with the OOPE being 14 times higher in private facilities for vaginal delivery and about 8 times higher for CS.

**Table 2:** Average OOPE by delivery and institution type.

Total OOPE	Vaginal	Caesarean
**Public**	₹ 1070.34	₹ 4187.22
**Private**	₹ 14849.48	₹ 33507.53

*Source:* calculated from NFHS-5.

### 4.4. Results for multivariate logistic regression at the national level

The results for the logistic regressions at the national level for both NFHS-4 and NFHS-5 are given in Table 3. We found that all major socioeconomic factors: education, wealth and place of residence (rural/urban) significantly determine the CS rate. The Odds ratio for CS if the place of residence is Urban was 1.18 times when the rural areas were the reference category. This is corroborated by the higher rates of CS in Urban areas. The higher odds could be explained by greater availability of medical institutions and thus higher institutional births. In major cities, institutional births are almost universal [54]. Similarly, for education, an increased likelihood of having CS is found as the level of education of the mother increases, in both versions of the NFHS. The odds of CS in NFHS-5 were 1.17 times higher if the mother had primary education, 1.72 times higher if she had secondary education, and 2.28 times higher if she had higher education, with no education serving as the reference category. A similar trend was also found in NFHS-4. Two contrasting assertions could be made about this variation in CS rates by education level. First, the vast economic literature indicates that higher education is linked with higher levels of income and wealth, which in this case could afford one access to medical institutions [55]. Second, there is evidence that indicates that affluent and highly educated women might opt for an elective CS because of perceived lower risk and pain [56]. Further, closely related to education, family wealth is also a major determinant of CS in both versions of the NFHS, with the odds of CS in NFHS-5 being 1.57 times for the poor when the poorest is the reference category. These odds of CS rates have been continuously increasing with an increase in wealth quintiles. This is explained by the fact that wealth brings better access to medical institutions, and often, those with higher wealth, for convenience and comfort, choose to go to private hospitals, which have much higher rates of CS. Finally, when considering the institutional factor behind CS, i.e., the public and private nature of the place of delivery, the odds ratio for a birth through CS in a private institution was 4.11 times that of a public institution, which served as the reference category. The odds for CS in NFHS-5 for private institutions in relation to public institutions have increased compared to NFHS-4. The factors responsible for this disparity in CS rates in Public and Private institutions have been extensively discussed in Section 1.4.

**Table 3:** Multivariate logistic regression results for India (NFHS-4 & NFHS-5).

Factors	INDIA					
	NFHS-4			NFHS-5		
Variable	Odds Ratio	P > |z|	conf. interval	Odds Ratio	P > |z|	conf. interval
Place of Delivery						
Public	1 [Reference]			1 [Reference]		
Private	3.70**	0.000	(3.58–3.82)	4.11**	0.000	(3.98–4.24)
Wealth Index						
Poorest	1 [Reference]			1 [Reference]		
Poorer	1.46**	0.000	(1.37-1.55)	1.57**	0.000	(1.50–1.65)
Middle	2.18**	0.000	(2.05–2.31)	2.19**	0.000	(2.08–2.23)
Richer	2.47**	0.000	(2.32–2.64)	2.38**	0.000	(2.25–2.50)
Richest	2.51**	0.000	(2.34–2.69)	2.28**	0.000	(2.14–2.41)
Education						
No Education	1 [Reference]			1 [Reference]		
Primary	1.21**	0.000	(1.14–1.29)	1.17**	0.000	(1.10–1.24)
Secondary	1.67**	0.000	(1.60–1.75)	1.72**	0.000	(1.64–1.80)
Higher	2.16**	0.000	(2.03–2.29)	2.28**	0.000	(2.16–2.41)
Place of Residence						
Rural	1 [Reference]			1 [Reference]		
Urban	1.18**	0.000	(1.13–1.23)	1.18**	0.000	(1.13–1.23)
Constant	0.04**	0.000	(0.04–0.05)	0.06**	0.000	(0.05–0.06)

Source: NFHS-5.

Confidence intervals are presented in parentheses. **p < 0.05 indicates statistical significance at the 5% level.

### 4.5. Results for multivariate logistic regression for selected states

To gain a more nuanced image of the factors driving CS rates in the country, logistic regressions were also performed for four selected states: Bihar, Uttar Pradesh, Tamil Nadu and Andhra Pradesh. The reasons for selecting these states are as follows: First, all four states are among the larger states by population. Second, the two groups of states differ extensively in terms of access to medical infrastructure. This disparity is evident in the vast difference between the four states on two key public health metrics: population per doctor and population served per bed in public facilities. Finally, the CS rates, as shown in Table 1, differ significantly for the four states. In Bihar and Uttar Pradesh, the CS rates are 9.57% & 12.31% respectively, which are clustered around the lower limit of the “WHO recommended” CS rate and lower than the national CS rate. While Tamil Nadu and Andhra Pradesh are among the states with CS rates above 40%. Further, Bihar and Uttar Pradesh are among the few large states with still considerably lower rates of institutional births, at 77.41% and 83.46%, respectively. In contrast, Tamil Nadu and Andhra Pradesh are states with some of the highest rates of institutional births, 99.43% and 96.75% respectively. It can be seen from Table 4 that in Bihar and Uttar Pradesh, access to both medical doctors and hospitals is highly limited. However, for Tamil Nadu and Andhra Pradesh, there is much better access to both Doctors and a relatively high availability of beds. These differences in medical facilities serve as a natural counterfactual, helping us better assess the true drivers of the high CS rate.

**Table 4:** State-wise key public health access indicators.

State	Population per doctor*	Population per bed ^	Institutional Birth (%) ^#^
Bihar	3356	5849	77.41%
Uttar Pradesh	3692	3427	83.46%
Tamil Nadu	696	994	99.60%
Andhra Pradesh	659	815	96.75%
India	1445	1638	86.43%

Source: * Department of Health and Family Welfare, Ministry of Health and Family Welfare [57].

^Calculated from 2011 Census and Ministry of Health and Family Welfare [58,59].

#Calculated from NFHS-5.

The results for the logistic regressions for Bihar and Uttar Pradesh are reported in Table 5. The results for both states are analogous to the results observed at the national level, with both socioeconomic and Institutional factors (Place of delivery) significantly influencing CS rates. In Uttar Pradesh, the odds of giving birth through CS in urban areas were 1.22 times higher than in rural areas, which served as the reference category. However, in Bihar, the odds were reversed, with the odds of birth through CS in Urban areas being only 0.76 times that in rural areas, which eludes conventional understanding. For both states, the probability of CS increases as maternal education increases, women with higher education were 1.61 times more likely to get a CS than those with no education in Bihar. The odds ratio in Uttar Pradesh is comparatively higher than in Bihar, women with higher education being 2.14 times more likely to give birth through CS compared to those with no education. The economic status of the family (*wealth index*) also significantly influences CS rates, with Women in the middle wealth quintile being 1.34 times more likely to undergo CS in Bihar, with this likelihood increasing to 1.58 times for those in the highest quintile compared to the poorest quintile. The pattern is similar in Uttar Pradesh, where the odds ratios are 1.38 for the middle quintile and 1.81 for the highest quintile, compared to the poorest quintile. Finally, the most dramatic result is the variation in CS rates between Public and Private institutions. The odds of giving birth through CS in a Private institution were 16.19 times that in a public institution in Bihar. Similarly, the odds of giving birth through CS in a Private institution were 8.62 times that in a public institution in Uttar Pradesh. This is a large difference when compared to the national measures, with the odds in Bihar being quadruple and in Uttar Pradesh double that at the national level. However, all other socioeconomic factors had similar odds at the national level and in Bihar and UP. This suggests that factors beyond purely medical considerations are driving CS rates in Bihar and UP. The very high magnitude of difference between Public and Private institutions clearly implicates that Private hospitals, driven by monetary incentives, prioritise CS over vaginal deliveries.

**Table 5:** Multivariate logistic regression results for Bihar and Uttar Pradesh.

Factors	NFHS-5					
	Bihar			Uttar Pradesh		
Variable	Odds Ratio	P > |z|	conf. interval	Odds Ratio	P > |z|	conf. interval
Place of Delivery						
Public	1 [Reference]			1 [Reference]		
Private	16.19**	0.000	(13.76–19.05)	8.62**	0.000	(7.84–9.47)
Wealth Index						
Poorest	1 [Reference]			1 [Reference]		
Poorer	1.16	0.093	(0.98–1.38)	1.31**	0.000	(1.14–1.50)
Middle	1.34**	0.005	(1.09–1.64)	1.38**	0.000	(1.19–1.60)
Richer	1.53**	0.000	(1.20–1.94)	1.84**	0.000	(1.58–2.14)
Richest	1.58**	0.004	(1.15–2.16)	1.81**	0.000	(1.52–2.14)
Education						
No Education	1 [Reference]			1 [Reference]		
Primary	1.18	0.147	(0.94–1.49)	1.17	0.057	(0.99–1.37)
Secondary	1.18**	0.047	(1.00–1.39)	1.55**	0.000	(1.38–1.74)
Higher	1.61**	0.000	(1.28–2.03)	2.14**	0.000	(1.86–2.45)
Place of Residence						
Rural	1 [Reference]			1 [Reference]		
Urban	0.76**	0.021	(0.60–0.96)	1.22**	0.001	(1.08–1.37)
Constant	0.03**	0.000	(0.02–0.03)	0.03**	0.000	(0.02–0.03)

Source: NFHS-5.

Pseudo R^2^ for Bihar = 0.27; Pseudo R^2^ for Uttar Pradesh = 0.21: Confidence intervals are presented in parentheses. **p < 0.05 indicates statistical significance at the 5% level.

The logistic regression results for Tamil Nadu and Andhra Pradesh reported in Table 6 present a considerably different situation. Unlike the National measures and the aforementioned state-level patterns for Bihar and UP, socioeconomic factors do not appear to be significantly influencing the odds of giving birth through CS in Tamil Nadu and Andhra Pradesh. This is evident from the fact that none of the socioeconomic factors, such as place of residence, wealth quintile, and level of education of the mother, are statistically significant in Tamil Nadu. Similarly, for Andhra Pradesh, most socio-economic factors do not significantly influence the odds of giving birth through CS. Only statistically significant result is for women with higher education; they are 1.68 times more likely to give birth through CS compared to women with no education. The only factor that remains a significant determinant of the odds of giving birth through CS in both Tamil Nadu and Andhra Pradesh is the institutional factor. With the odds of giving birth through CS in Private institutions being 3.08 times that in public facilities in Tamil Nadu, this is, however, lower than the odds ratio of 4.11 at the national level. Similarly, for Andhra Pradesh, the odds of giving birth through CS in private hospitals were 4.16 times higher than in public institutions.

**Table 6:** multivariate logistic regression results for Tamil Nadu and Andhra Pradesh.

Factor	NFHS-5					
	Tamil Nadu			Andhra Pradesh		
Variable	Odds Ratio	P > |z|	conf. interval	Odds Ratio	P > |z|	conf. interval
Place of Delivery						
Public	1 [Reference]			1 [Reference]		
Private	3.08**	0.000	(2.70–3.52)	4.16**	0.000	(3.40–5.09)
Wealth Index						
Poorest	1 [Reference]			1 [Reference]		
Poorer	1.07	0.736	(0.73–1.57)	1.35	0.298	(0.76–2.39)
Middle	1.12	0.551	(0.77–1.64)	1.59	0.103	(0.91–2.77)
Richer	1.15	0.482	(0.78–1.68)	1.35	0.315	(0.75–2.41)
Richest	0.99	0.957	(0.66–1.48)	1.75	0.085	(0.92–3.32)
Education						
No Education	1 [Reference]			1 [Reference]		
Primary	1.4	0.212	(0.82–2.38)	0.94	0.746	(0.64–1.38)
Secondary	1.06	0.802	(0.65–1.73)	1.39	0.052	(1.0–1.94)
Higher	1.31	0.284	(0.80–2.16)	1.68**	0.011	(1.13–2.52)
Place of Residence						
Rural	1 [Reference]			1 [Reference]		
Urban	0.99	0.937	(0.86–1.14)	1.09	0.503	(0.84–1.43)
Constant	0.46**	0.009	(0.26–0.82)	0.03**	0.000	(0.11–0.33)

Source: NFHS-5.

Pseudo R^2^ for Tamil Nadu = 0.05; pseudo R^2^ for Andhra Pradesh = 0.11; Confidence intervals are presented in parentheses. **p < 0.05 indicates statistical significance at the 5% level.

## 5. Discussion

The common theme among all states has been that CS rates are greatly influenced by factors other than purely medical ones, which ideally shouldn’t be the case. The decision whether a birth needs a CS is a purely medical one, and socioeconomic or institutional factors should not influence it. However, we found that the odds of CS were significantly determined by socioeconomic factors only in states like Bihar and UP, where the medical infrastructure is underdeveloped, the affordability of medical services is limited due to the lower economic status of these states, and consequently, a substantial proportion of births still occur outside of medical institutions. Contrarily, these socioeconomic factors do not seem to influence the odds of CS as strongly in states like Tamil Nadu and Andhra Pradesh, which are states with some of the best public medical infrastructure in the country, with higher ability to access these medical facilities on the grounds of better access to these medical facilities due to better economic conditions.

The results help us answer a critical question that has confounded us as to what really is causing the high rate of CS. This study seeks to determine whether private hospitals driven by profit motives and their power to exploit informational asymmetry, induce demand for unnecessary CS, or whether it is affluent individuals with higher education and economic status who demand CS for perceived lower risks and pain, and Private facilities fulfil this demand. Our results suggest a contrary explanation, as evident from the astronomically higher odds of CS in private institutions compared to Public Ones, irrespective of the state, and the fact that the odds for the institutional factor were also greater by magnitudes than any other socioeconomic factor. Private institutions are the single largest *(non-medical)* determinant of whether a child is born through CS or vaginal delivery. This finding aligns with the theoretical framework of Physician-Induced Demand, which posits that information asymmetry between healthcare providers and patients enables suppliers to influence demand for medical services beyond what is medically necessary [40,42].

Moreover, while assessing the claim that private hospitals are not responsible for the high rates of CS, rather, it is just catering to the demand for CS. The results of the state-level logistic regressions further weaken the argument, as if socioeconomic factors were the determining factor of CS, they should significantly influence the odds of CS universally, i.e., across all states. This, however, is not found to be true, as in states like Tamil Nadu and Andhra Pradesh, where the medical infrastructure is well-developed, socioeconomic factors do not significantly determine the odds of CS. Similar regional disparities in CS rates across Indian states have been documented by Neethi Mohan et al. (2023), who observed variations between Tamil Nadu and Chhattisgarh, with institutional factors playing a predominant role in determining CS rates [47]. Whatever the determining factor is, it should be consistently significant across states; socioeconomic factors clearly are not. The only factor that remains consistently significant across states is the institutional setting of the birth, i.e., whether the birth occurred at a public or private institution.

If that is the case, we must also answer why socioeconomic factors significantly influence the odds of giving birth through CS in Bihar, UP and at the national level. This can be understood by examining the mechanism through which socioeconomic factors influence access to medical institutions. In states like Bihar and UP, where medical infrastructure is scarce and access is difficult, people with better socioeconomic conditions are the ones who can access these medical facilities and thus end up having significantly higher odds of CS than those who cannot afford access to these medical institutions. Within these states, those with the highest socioeconomic status often choose private institutions for convenience and perceived higher standards; however, because private institutions are driven by profit incentives, they end up with much higher odds of CS. Economic incentives in private healthcare settings have been shown to systematically influence clinical decision-making, with facilities facing higher reimbursement rates for CS demonstrating correspondingly higher CS rates [42].

This, however, does not hold in the case of states like Tamil Nadu and Andhra Pradesh, where access to medical institutions is almost universal due to greater availability of both public and private institutions. Therefore, one’s socioeconomic status does not dictate access to these institutions; thus, socioeconomic factors no longer significantly determine the odds of CS. The only factor that continues to significantly determine the CS odds ratio is whether one goes to a public or private institution. Even in states like Tamil Nadu and Andhra Pradesh, the nature of private institutions and the incentives driving them remain the same: to maximise their profits. Thus, those in these states who end up attending private institutions have much higher odds of CS. To conclude, socioeconomic factors are determining factors of CS only as long as they determine access to medical facilities. However, it is indeed the institutional setting that needs to be implicated for the high and increasing rate of CS in the country.

However, as asserted by Rayhan (2020), socio-economic factors might very well influence CS rates indirectly, which might not be correctly attributed to the coefficients in our regression, and some of the effects of CS might get subsumed under the place of delivery (institutional setting) variable, wrongfully attributing some of the impact of socioeconomic factors to the institutional factor [60]. This could be explained by the fact that even when healthcare access is universal, individuals with higher socioeconomic status could afford better hospitals, and better and more personalised care, most of it would be available in a private hospital, and some of the high rates of CS in private hospitals might be explained by the higher demand for it in high socio-economic groups. However, the assertion made by Rayhan (2020), that place of delivery is only a “*proximate determinant of CS rather than a strong predictor”* can’t be entirely accurate, as private hospitals consistently have high odds of CS across states, moreover these higher odds continue to hold even when other socioeconomic factors don’t hold, and assuming that place of delivery is only a proximate factor would be ignoring the existence of large monetary incentives for these clinics and to deny the effect of well-established theoretical models like that of Physician Induced Demand [40]. Given the limited data available, it is indeed impossible to establish a causal link, however at the same time one can look at the data, the theoretical models that govern rational human behaviour, the incentives in place and the extensive qualitative work that has been done on this issue and deduce with considerable certainty that institutional setting (public/private) is one of the largest determinants of whether a child is born through CS or not, and thus it becomes imperative to implicate the Private institutions and to bring about policy and oversight to keep in check the perverse incentives governing private practices.

## 6. Conclusion and policy implications

The substantial and sustained increase in CS rates in India is a major public health concern. Our analysis confirms that the increase in CS rates is not merely a reflection of medical necessity but is significantly influenced by non-medical factors, particularly socioeconomic and institutional factors. The findings reveal that while socioeconomic factors, such as the mother’s level of education, economic status, and place of residence, generally increase the likelihood of birth through CS. However, the most significant factor determining the likelihood of CS across states and at the national level is the type of medical facility (public or private). This aligns with the theories of Physician-induced demand and the significant financial incentives in the private facilities, driving higher rates of CS. Even when socioeconomic factors influence CS rates, we assert that they do so by determining access to private institutions, as those with higher socioeconomic status can afford private healthcare and thus are subjected to a higher likelihood of birth through CS.

Our study highlights the urgent need for a dual-policy approach in India, one that promotes access to medically necessary CS in underserved regions and another that curbs the rising trend of unnecessary CS, particularly in private facilities. States with lower CS rates are recommended to improve medical facilities and their accessibility. This includes investment in maternal health services and increased staffing and capacity in healthcare institutions. This would enable these regions to reach the ideal level of CS for better health outcomes through reducing NMR and MMR. However, for states with very high CS rates, the policy should be targeted at rationalising the CS rate; interventions on both the supply and the demand side could achieve this. On the demand side, a crucial step is to ensure universal prenatal assessment of each pregnancy to determine the risk level of the pregnancy. Additionally, awareness programs and providing parents with the risk profile of the pregnancy beforehand could reduce the informational asymmetry that private hospitals exploit. On the supply side, multiple policy interventions like centralised registration of mode of delivery of birth and auditing of hospitals with consistently elevated rates of CS, increasing the number of OB-GYNs and SBAs, particularly medically trained midwives who would be responsible for low-risk pregnancies (European model), and sensitisation, retraining of doctors should be undertaken.

Moreover, there is an urgent need to improve the recording of birth data. Expanding key indicators under the Reproductive and Child Health (RCH) portal and ensuring that all institutional births are accurately logged will provide policymakers with more accurate and granular insights. Secondly, the Ayushman Bharat Digital Health Mission (ABDM) should be leveraged to integrate delivery records with Ayushman Bharat Health Accounts (ABHA ID), enabling centralized birth registration, real-time monitoring for state and district health authorities, and anonymized datasets for researchers to analyse trends and root causes. While these policy measures are both relevant and feasible, implementation may face several challenges. Data integration under ABDM could encounter resistance from stakeholders concerned about privacy and centralization. Auditing of private hospitals may be opposed by vested commercial interests, especially where regulation threatens profit margins. Midwifery-led models will require significant regulatory and institutional changes, as well as long-term investments in training and infrastructure. Moreover, awareness campaigns must be carefully designed to avoid stigmatizing medically indicated CS or creating fear among mothers. Addressing these barriers will require coordinated efforts among state governments, healthcare providers, and civil society, alongside feedback mechanisms to ensure that interventions are effective and equitable.

## 7. Limitations

This study has several limitations that should be acknowledged. First, as a cross-sectional analysis, we cannot establish causal relationships between institutional factors and CS rates, only associations. Second, the NFHS data do not include detailed clinical indications for each CS, limiting our ability to distinguish between medically necessary and potentially unnecessary procedures. Third, while we controlled for major socioeconomic factors, unmeasured confounders such as patient preferences, physician characteristics, or facility-specific protocols may influence the results. Fourth, the five-year recall period for births in NFHS may be subject to recall bias, though this is likely minimal for major events like mode of delivery. Finally, our state-level analysis was limited to four states due to data availability and analytical focus, which may not fully represent the diversity across all Indian states. Despite these limitations, the large, nationally representative sample and robust analytical approach provide valuable insights into CS patterns in India.

## 8. Future research

As NFHS-6 data becomes available over the next year, it will offer a timely opportunity to revisit and update this analysis. A comparative study with NFHS-4 and NFHS-5 can help assess how CS trends have evolved in the post-pandemic period, particularly in light of the COVID-19 lockdowns and disruptions to institutional delivery services. Another promising direction is to explore how the expanded coverage of the Ayushman Bharat health insurance scheme has influenced access to private healthcare and whether this has contributed to changes in CS rates. Additionally, future studies could examine the long-term maternal and child health outcomes associated with CS births using cohort or linked administrative data. Finally, collaborations with state governments or private healthcare providers to conduct randomized controlled trials (RCTs) assessing the impact of interventions such as midwifery-led care models or universal prenatal screening could yield valuable evidence to inform policy design and scale-up.

## Supporting information

S1 Table State abbreviated names for India. (DOCX)

## Abbreviations

ABDM: Ayushman Bharat Digital Health Mission
ABHA ID: Ayushman Bharat Health Accounts ID
ANC: Antenatal Care
ASHA: Accredited Social Health Activist
AUC: Area Under ROC Curve
BMI: Body Mass Index
CI: Confidence Interval
CS: Caesarean Section
DHS: Demographic and Health Survey
GDP: Gross Domestic Product
NFHS: National Family Health Survey
NMR: Neonatal Mortality Rate
MMR: Maternal Mortality Rate
OB-GYN: Obstetrician-Gynaecologist
OOPE: Out-of-Pocket Expenditure
OR: Odds Ratio
PHC: Primary Health Centre
RCH: Reproductive and Child Health
RCT: Randomised Control Trial
WHO: World Health Organization
