# Supplementary material for: Caesarean section epidemic in India: Is private sector to blame? A multivariate logistic regression analysis of the National Family Health Survey
Source: PLoS One. 2026 Jul 6;21(7):e0352156. doi: 10.1371/journal.pone.0352156 (PMC13336216; doi:10.1371/journal.pone.0352156)
Supplement: S1 Table — (DOCX) [file pone.0352156.s001.docx]

| Abbreviated names | States name |
| --- | --- |
| AN | Andaman & Nicobar |
| AP | Andhra Pradesh |
| AR | Arunachal Pradesh |
| AS | Assam |
| BR | Bihar |
| CG | Chhattisgarh |
| CH | Chandigarh |
| DL | Delhi |
| GJ | Gujarat |
| GO | Goa |
| HP | Himachal Pradesh |
| HR | Haryana |
| JH | Jharkhand |
| JK | Jammu & Kashmir |
| KA | Karnataka |
| KL | Kerala |
| MH | Maharashtra |
| ML | Meghalaya |
| ML | Manipur |
| MP | Madhya Pradesh |
| MZ | Mizoram |
| NL | Nagaland |
| OD | Odisha |
| PU | Punjab |
| PY | Puducherry |
| RJ | Rajasthan |
| SK | Sikkim |
| TG | Telangana |
| TN | Tamil Nadu |
| TR | Tripura |
| UK | Uttarakhand |
| UP | Uttar Pradesh |
| WB | West Bengal |

Appendix 1: State Abbreviated names for India.
